# Loss of age-accumulated *crh-1* circRNAs ameliorate amyloid β-induced toxicity in a *C. elegans* model for Alzheimer’s disease

**DOI:** 10.3389/fnagi.2025.1464015

**Published:** 2025-03-24

**Authors:** Hussam Z. Alshareef, Thomas Ballinger, Everett Rojas, Alexander M. van der Linden

**Affiliations:** Department of Biology, University of Nevada, Reno, NV, United States

**Keywords:** Alzheimer’s disease model, Aβ_1–42_, *crh-1*, CREB, circular RNA, collagen, *C. elegans*, aging

## Abstract

Circular RNAs (circRNAs) are non-coding RNAs mostly derived from exons of protein-coding genes via a back-splicing process. The expression of hundreds of circRNAs accumulates during healthy aging and is associated with Alzheimer’s disease (AD), which is characterized by the accumulation of amyloid-beta (Aβ) proteins. In *C. elegans*, many circRNAs were previously found to accumulate during aging, with loss of age-accumulated circRNAs derived from the CREB gene (circ-*crh-1*) to increase mean lifespan. Here, we used *C. elegans* to study the effects of age-accumulated circRNAs on the age-related onset of Aβ-toxicity. We found that circ-*crh-1* mutations delayed Aβ-induced muscle paralysis and lifespan phenotypes in a transgenic *C. elegans* strain expressing a full-length human Aβ-peptide (Aβ_1–42_) selectively in muscle cells (GMC101). The delayed Aβ phenotypic defects were associated with the inhibition of Aβ aggregate deposition, and thus, genetic removal of circ-*crh-1* alleviated Aβ-induced toxicity. Consistent with a detrimental role for age-accumulated circRNAs in AD, the expression level of circ-*crh-1* expression is elevated after induction of Aβ during aging, whereas linear *crh-1* mRNA expression remains unchanged. Finally, we found that the delayed onset of Aβ-induced paralysis observed in circ-*crh-1* mutants is dependent on the *col-49* collagen gene. Taken together, our results show that the loss of an age-accumulated circRNA exerts a protective role on Aβ-induced toxicity, demonstrating the utility of *C. elegans* for studying circRNAs in AD and its relationship to aging.

## 1 Introduction

Circular RNAs (circRNAs) emerged as an intriguing class of non-coding RNAs with unique closed-loop structures. CircRNAs are generated through a process known as back-splicing during conventional RNA splicing, where the 3′ and 5′ ends of a pre-mRNA molecule are covalently bonded, yielding a circular configuration (Li et al., 2018). Most identified circRNAs are produced from exons of protein-coding genes (Zhang et al., 2014). Their lack of free ends confers circRNAs a degree of resistance to exoribonuclease digestion compared to their linear counterparts (Jeck et al., 2013), which can contribute to their stability and abundance. Despite these characteristics, the functions of many circRNAs remain largely elusive, although their functions appear to be intertwined with the molecules they interact with.

CircRNAs accumulate during the normal process of aging in *C. elegans* (Cortés-López et al., 2018), *Drosophila* (Westholm et al., 2014; Hall et al., 2017), and mice (Gruner et al., 2016) and are found to play both positive and negative roles in the aging of *C. elegans*, *Drosophila*, and various mammalian tissues (Knupp and Miura, 2018; Kim et al., 2021). Prominent examples include the extension of lifespan through *circSfl* transgenic overexpression in *Drosophila* (Weigelt et al., 2020) and the loss of the circRNA derived from the host gene *crh-1*/CREB in *C. elegans* (Knupp et al., 2022). Additionally, growing evidence suggests key roles of circRNAs in Alzheimer’s disease (AD) by affecting mechanisms such as neuroinflammation, oxidative stress and autophagy, as well as amyloid-beta (Aβ) production and degradation (Beylerli et al., 2024). For example, circular RNA *ciRS-7* (also known as *CDR1as*) inhibits the activity of microRNA, *mir-7* (Hansen et al., 2013), which subsequently effects the accumulation of Aβ plaques in AD (Shi et al., 2017; Sun et al., 2023). Considering that brain aging is highly associated with AD pathogenesis, circRNAs that accumulate with aging may also contribute to AD. CircRNAs can act as molecular sponges, sequestering microRNAs (miRNAs) (Hansen et al., 2013; Zhang et al., 2020) and RNA-binding proteins (RBPs) (Patop et al., 2019; Chen, 2020) away from their messenger RNA (mRNA) targets, thereby altering the splicing or expression patterns of these mRNAs. However, our understanding of the functional roles of age-accumulated circRNAs in AD remains limited.

*C. elegans* presents a powerful model organism for studying age-associated circRNAs in AD. Previously, we demonstrated that a majority of circRNAs expressed in *C. elegans* accumulate during aging (Cortés-López et al., 2018). Using CRISPR/Cas9, we genetically removed two abundant age-accumulated circRNAs derived from the *crh-1* gene (circ-*crh-1*), which encodes the homolog of CREB, without disrupting the linear RNA and its associated activated protein (Knupp et al., 2022). The genetic loss of this age-accumulated circ-*crh-1* extended the mean lifespan of *C. elegans* (Knupp et al., 2022), suggesting that circ-*crh-1* abundance might contribute to age-related decline. Here, we extend our findings by testing the impact of circ-*crh-1* removal on a severe model of inducible amyloidosis in *C. elegans*. We used the transgenic *C. elegans* strain (GMC101), which expresses human Aβ_1–42_ peptides constitutively in muscle cells that mimics the pathological features of AD (McColl et al., 2012). We found that the loss of circ-*crh-1* expression delayed Aβ-induced paralysis of GMC101, improved its shortened mean lifespan, and reduced Aβ aggregate formation. Moreover, circ-*crh-1*(-) mutants exhibited an increase in the expression of the *col-49* collagen gene. Mutations in *col-49* exacerbated Aβ-induced paralysis, while *col-49* overexpression reduced paralysis induced by Aβ. Further investigation revealed that the delayed onset of Aβ-induced paralysis observed in circ-*crh-1*(-) mutants is dependent on *col-49* expression. Together, our results show that loss of age-accumulated *chr-1* circRNAs increase the expression of the *col-49* collagen gene, thereby reducing Aβ-induced toxicity in a *C. elegans* transgenic model for Alzheimer’s disease.

## 2 Materials and methods

### 2.1 Strains, general animal cultivation and genetic controls

Worms were cultivated on the surface of NGM agar seeded with the *Escherichia coli* strain OP50 as the primary food source and grown in 20°C incubators using standard protocols unless indicated otherwise. All experiments were performed on hermaphrodites. The wild-type strain N2, variety Bristol (Brenner, 1974) and other strains used in this study are listed in Supplementary Table 1. Strains were constructed using standard genetic methods (Fay, 2006) and genotypes were confirmed either by phenotype (for example, the transgenic strain was marked by fluorescence) or by PCR (for example, by identifying small deletions in mutant strains).

### 2.2 Generation of plasmids, transgenic animals, and *col-49* mutants

To generate transgenic worms expressing the circ-*crh-1* in muscle cells, exon 4 of *crh-1* and intronic sequences flanking exon 4 (Figure 4A) were cloned into the pMC10 plasmid (a kind gift from the Sengupta Lab). Next, promoter sequences of *myo-3* (∼2.5 kb) were cloned at the 5′-end of the circ-*crh-1* sequence using the multi cloning site (MCS) of pMC10. The generated *myo-3p::circ-crh-1* construct along with the *unc-122p::RFP* co-injection marker (AddGene) was injected into VDL1300 *crh-1(syb385); dvIs100[unc-54p::A*β_1–42_*::unc-54 3*′*-UTR*, *mtl-2p::GFP*] animals to create the VD12 strain. Transgenic worms carrying extrachromosomal arrays overexpressing *pie-1p::circ-crh-1* (VDL975), *rab-3p::circ-crh-1* (VDL1104) were crossed with the VDL1300 strain to create VDL1306 and VDL1307 strains (Supplementary Table 1). A *col-49* mutant allele (*syb8747*) harboring a 1180bp deletion was generated using a Co-CRISPR method to create the VD10 strain (SunyBiotech), which was confirmed by PCR and Sanger sequencing. sgRNAs used to generate the *col-49(syb8747)* mutant were Sg1: 5′-cctcatcatcatgtggaaattcg and Sg2: 5′-cccacctagaactgcttgattcg. We crossed the VD10 with the GMC101 strain to create VDL1308 *col-49(syb8747); dvIs100[unc-54p::A*β_1–42_*::unc-54 3*′*-UTR*, *mtl-2p::GFP*]. We then crossed the VDL1308 with the VDL1300 strain to create VDL1310 *crh-1(syb385); col-49(syb8747); dvIs100[unc-54p::Aβ_1–42_::unc-54 3′-UTR, mtl-2p::GFP*] using standard genetic methods. We generated a transgenic line that overexpresses *col-49* from a transgene carrying a multiple copy array of the *col-49* genomic sequence (∼1.2 kb) under control of its endogenous promoter upstream sequence (∼2 kb) and *col-49* 3′-UTR sequence (∼1 kb). The resulting plasmid (*col-49p::col-49 genomic::col-49 3′-UTR*) along with the *unc-122p::RFP* co-injection marker was injected into wild-type worms to generate VD16 (SunyBiotech). Next, we crossed the VD16 with the GMC101 strain to create the VDL1309 strain using standard genetic methods. Supplementary Table 1 shows all strains created and used in this study.

### 2.3 Lifespan analysis

All strains were maintained at 20°C for at least two generations before the lifespan assay. Adult worms age-synchronized by hypochlorite treatment and collected eggs were hatched overnight at 20°C. L1 larvae were then plated onto NGM plates seeded with *E. coli* OP50 bacteria. At the L4 larval stage, 90–150 worms per genotype were transferred to new 6 cm NGM plates seeded with 10x concentrated *E. coli* OP50 bacteria containing 0.5 μM 5-fluorodeoxyuridine (FUdR) to inhibit the development of self-progeny, and then shifted at the young adult stage to 25°C. Each strain was assayed in parallel and each plate contained 10–15 worms. Worms were blindly scored every day and were considered dead when they did not respond to touch of the platinum wire pick and were subsequently removed from the plate. Worms that experienced ventral rupture, bagging, or walling were censored from the lifespan analysis.

### 2.4 Paralysis assays

The paralysis assay was performed using GMC101 transgenic animals expressing *unc-54p::A*β_1–42_ as described previously (McColl et al., 2012). Briefly, worms were age-synchronized by hypochlorite bleaching and cultivated at 20°C. After they reached the L4 larval stage, worms were transferred to assay plates freshly seeded with *E. coli* OP50 bacteria, containing 0.5 μM of FUdR to inhibit the development of self-progeny, and then shifted at the young adult stage to the higher permissive 25°C temperature to induce paralysis unless indicated otherwise. About 15 worms were placed on each 6 cm NGM plate, and animals were blindly scored every 24 h as “paralyzed” if they failed to perform a full body wave propagation following a repeated touch-provoked response.

### 2.5 Total RNA collection and extraction

Worms were age-synchronized worms by hypochlorite treatment and collected eggs were hatched overnight at 20°C in 1x M9 buffer. L1 larvae were then plated onto NGM plates seeded 10x concentrated *E. coli* OP50 bacteria and allowed to develop to the L4 larval stage at 20°C. L4 larvae were then collected, washed and re-plated onto *E. coli* OP50 seeded NGM plates containing 0.5 μM FUdR. Worms were either upshifted to 25°C or kept at 20°C. Adult worms were collected at different aging time-points and washed with 1x M9 buffer through 35 μM nylon mesh to remove bacteria. Worm pellets of 100–300 μl were then transferred into green RINO tubes (Next Advance) and TRizol LS reagent (ThermoFisher Scientific, Cat #10296028) was added in a 1:3 ratio. Worms were immediately lysed by bead beating them for 5 min using a Bullet Blender Pro Storm (Next Advance). Total RNA was extracted using the Purelink RNA mini-kit, followed by a DNAse I treatment following the manufacturer’s protocol (Ambion, Cat #12183020). RNA was quantified by a Nanodrop. Bioanalyzer or tapestation (Agilent) were used for qualification as needed. and samples were stored at −80°C.

### 2.6 Analysis by RT-qPCR

To quantify and confirm individual circular or linear transcripts, 0.5 μg total RNA was reverse transcribed using Superscript III to prepare cDNA using random hexamers (Invitrogen, Cat #18080051). Next, cDNA samples were diluted and used with PowerUp SYBR Green Master Mix (Applied Biosystems, Cat #A25471) for RT-qPCR analysis analyzed on a CFX96 Real-Time System (Bio-Rad). For RT-qPCRs of circRNAs, we used outward-facing primers. For host gene linear RNA counterparts, one primer was located in the circularizing exon and the other was located in the upstream or downstream non-circularizing exon. For linear mRNAs such as collagen-encoding genes and Aβ mRNA, we used forward-facing primers. Fold-change values were calculated using wild-type (N2) ΔCt as control values for the 2^–ΔΔ*Ct*^ method. Data is normalized to housekeeping genes (*cdc-42* or *act-1*) mRNA. Primer sequences are listed in Supplementary Table 2.

### 2.7 Imaging and quantification of Aβ aggregates in living animals

Four-day old adults were randomly collected during paralysis assays and stained with 1 mM X-34 (Sigma, Cat # SML1954) 10 mM Tris-HCl pH 8.0 for 2 h as previously described (Link et al., 2001). Stained worms were then washed twice with 1x M9 and transferred to *E. coli* seeded NGM plates containing 0.5 μM FUdR for 24 h to de-stain worms. De-stained 5-day old adults were then placed onto a 2% agarose pad with 10 μM levamisole to anesthetize worms. Confocal microscopy was used to acquire and capture images of the worm head using a 40x oil objective (405 nm excitation, 470–520 nm emission range). ImageJ software (NIH) was used to quantify Aβ aggregates.

### 2.8 Total collagen level determination

As previously described (Teuscher et al., 2019), worms were age-synchronized by hypochlorite treatment and collected eggs were hatched overnight at 15°C, 20°C, or 25°C in 1x M9 buffer. L1 larvae were then plated onto NGM plates seeded *E. coli* OP50 bacteria and allowed to develop 24 h post-L4 larval stage (1-day adult) at 15°C, 20°C, or 25°C. 1-day adult worms were then collected with 1x M9 buffer and washed with dH_2_O through a 35 μM nylon mesh to remove bacteria. Worm pellets of 300 μl were then transferred into green RINO tubes (Next Advance) and lysed by bead beating them for 10 min using a Bullet Blender Pro Storm (Next Advance). Total collagen level was determined using the QuickZyme Total Collagen Kit (QuickZyme Biosciences), following the manufacturer’s protocol. Briefly, lysate samples were mixed with 12M HCl solution and incubated for 20 h at 95°C. Then assay buffer was added, and the 96-well plate was incubated at room temperature for 20 min, followed by the addition of the detecting reagent and incubation at 60°C for 60 min. Total collagen level was measured and quantified as a fraction of total protein abundance using a Synergy HT BioTek microplate reader. Total protein levels were quantified using the Pierce*™* BCA Protein Assay Kit (ThermoFisher Scientific) following the manufacturer’s protocol.

### 2.9 Statistical analysis

Statistical comparisons and graphical representations were performed with the Online Application for Survival Analysis, OASIS 2 (Han et al., 2016). For lifespan survival and paralysis curves, we used the Mantel-Cox log-rank test. Other data were analyzed using Graphpad Prism 9 software and statistical comparisons made include the Mann-Whitney *t*-test or the one-way ANOVA followed by a *post hoc* multiple-comparisons test. *p*-values are reported in the figure legends.

## 3 Results

### 3.1 circ-*crh-1* mutants delay the onset of Aβ-induced paralysis

**FIGURE 1:**
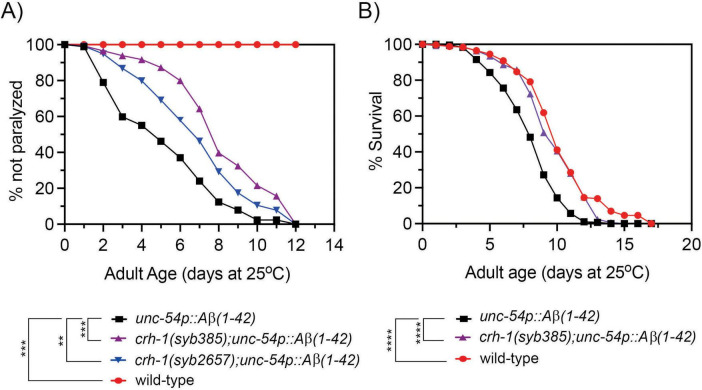
Loss of circ-*crh-1* delays paralysis and decreases the short lifespan of an Aβ-induced proteotoxicity model. **(A)** The onset of paralysis measured in the Aβ-proteotoxicity model strain, GMC101 (*unc-54p::A*β*_1–42_*) and the GMC101 strain carrying either a *syb385* mutation (purple) or *syb2657* mutation (blue) after a temperature upshift from 20°C to 25°C. Wild-type (red) or *unc-54p::A*β*_1–42_* (GMC101, black) animals at 20°C do not show paralysis. 3 independent trials with *n* > 140 animals for each assay and genotype in the presence of 0.5 μM FUdR. Asterisks indicate statistical significance with **p < 0.01, ***p < 0.001. **(B)** Lifespan curves for *unc-54p::A*β*_1–42_* (GMC101) animals compared to *crh-1(syb385); unc-54p::A*β*_1–42_* and wild-type animals at 25°C. Induction of *unc-54p::A*β*_1–42_* shortens lifespan compared to wild-type (****p < 0.0001, Mantel-Cox log-rank test), which can be reversed by *syb385* mutations. There is a non-significant difference in mean lifespan between *crh-1(syb385); unc-54p::A*β*_1–42_* and wild-type (*p* < 0.035, Mantel-Cox log-rank test). See Supplementary Table 3 for lifespan statistics. *n* = 3–4 independent lifespan assays were performed with *n* = 90–150 animals for each assay and genotype in the presence of 0.5 μM FUdR (see section “2 Materials and methods”).

We previously demonstrated that expression of circ-*crh-1* accumulates with *C. elegans* aging (Cortés-López et al., 2018) and that loss of circ-*crh-1* expression results in a significant extension of mean lifespan (Knupp et al., 2022). To directly test whether circ-*crh-1* expression plays a role in Aβ-induced toxicity, we used the GMC101 strain (further referred as *unc-54p::A*β_1–42_) in which expression of human Aβ_1–42_ peptides constitutively in muscle cells provokes an inducible age-progressive full-body paralysis when shifted from the non-restrictive 20°C temperature to a higher permissive 25°C temperature (McColl et al., 2012). We observed that mutations in circ-*crh-1* (*syb385* and *syb2657*), which exhibit a complete loss of circ-*crh-1* expression but normal linear *crh-1* expression, showed less severe paralysis in *unc-54p::A*β_1–42_ animals at the higher permissive 25°C temperature (Figure 1A; Knupp et al., 2022). Thus, loss of circ-*crh-1* expression significantly delayed the onset of Aβ-induced paralysis.

### 3.2 circ-*crh-1* mutants reduce Aβ-induced lifespan shortening

To further test the protective effect of circ-*crh-1* expression Aβ-induced toxicity, we next tested its loss on the lifespan of *unc-54p::A*β_1–42_ expressing animals. Previous work has shown that the expression of Aβ_1–42_ in body wall muscle cells severely decreased lifespan (Gallrein et al., 2021). Similarly, we found that *unc-54p::A*β_1–42_ expressing animals led to a significantly shorter mean lifespan compared to wild-type controls (20.5% reduction, 8.10 days for GMC101 versus 10.19 days for wild-type, *p* < 0.0001) when animals were shifted from 20°C to the higher permissive 25°C temperature (Figure 1B). Interestingly, *crh-1(syb385)* was able to restore the reduced lifespan of *unc-54::A*β_1–42_ animals back to wild-type levels (9.7 days for *crh-1(syb385); unc-54::A*β_1–42_ versus 8.1 days for GMC101, *p* < 0.0001) (Figure 1B). No significant differences were observed in mean lifespan between *unc-54::A*β_1–42_ and wild-type animals (*p* = 0.614) at the 20°C temperature (no Aβ-induction) (Supplementary Figure 1). Thus, circ-*crh-1* mutations prevented lifespan shortening induced by Aβ. Together, these findings suggest that loss of circ-*crh-1* expression protects *C. elegans* from Aβ-induced toxicity.

### 3.3 circ-*crh-1* expression is increased following Aβ-induction

**FIGURE 2:**
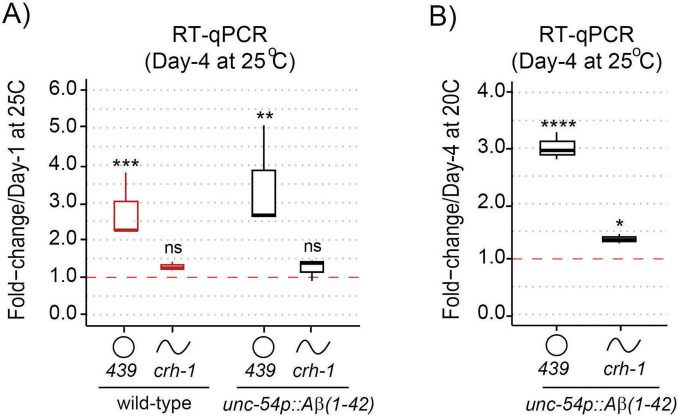
circ-*crh-1* expression is increased after Aβ-induction during aging. **(A)** RT-qPCR expression of the abundant circular circ-*crh-1* (*cel_circ_0000439*) and linear *crh-1* transcripts in 4-day adults normalized to 1-day adults of wild-type and *unc-54p::A*β*_1–42_* (GMC101) at 25°C. **(B)** RT-qPCR expression of circular and linear *crh-1* transcripts in day-4 adults of *unc-54p::A*β*_1–42_* (GMC101) at 25°C (after Aβ-induction) normalized to 1-day adults of *unc-54p::A*β*_1–42_* (GMC101) at 20°C (no Aβ-induction). circ-*crh-1* expression increases after induction of Aβ. *n* = 3 independent biological samples for both panels **(A)** and **(B)**. For RT-qPCR expression analysis, data in panels **(A)** and **(B)** was normalized to *cdc-42* mRNA. Data is represented as mean ± SEM. ns, not significant. *p < 0.05, **p < 0.01, ***p < 0.001, ****p < 0.0001.

We next examined the impact of Aβ-induction on the expression of the age-accumulated circ-*crh-1* in aged *unc-54::A*β_1–42_ animals compared to wild-type. We therefore conducted RT-qPCR analysis to measure the RNA fold change of circ-*crh-1* expression (i.e., *cel-circ_0000439*) in 4-day old adults compared to 1-day old adults for wild-type and between *unc-54::A*β_1–42_ animals shifted from 20°C to the higher permissive 25°C temperature (after Aβ-induction). As expected, the fold-change ratio of circ-*crh-1* gene expression was higher in *unc-54::A*β_1–42_ animals than wild-type by 1.25-fold (Figure 2A). Importantly, expression of linear *crh-1* was not significantly affected after Aβ-induction in 1-day and 4-day old adults (Figure 2A). Similar results were observed when normalizing circ-*crh-1* gene expression of 4-day old *unc-54::A*β_1–42_ adults at 25°C (after Aβ-induction) to the 20°C temperature (before Aβ-induction) (Figure 2B). These results suggest that Aβ-induction positively regulates circ-*crh-1* expression, consistent with a detrimental role for age-accumulated circRNAs in AD.

### 3.4 Loss of circ-*crh-1* expression reduces Aβ-aggregation

**FIGURE 3:**
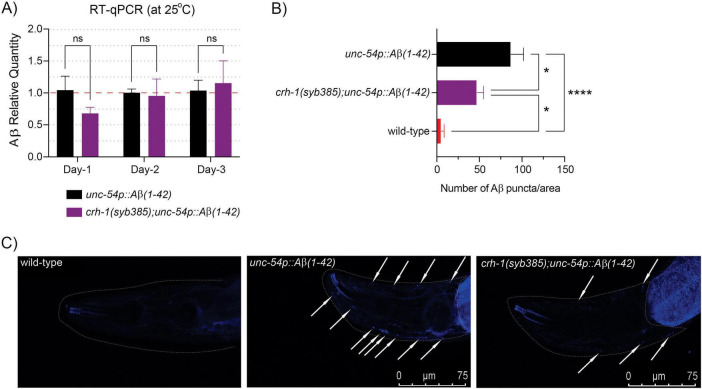
circ-*crh-1* mutation reduces Aβ-aggregation. **(A)** Relative quantity of Aβ_1–42_ gene expression in *unc-54p::A*β_1–42_ (GMC101, black), *crh-1(syb385); unc-54p::A*β_1–42_ (purple) animals during aging (1-day, 2-day, and 3-day old adults at 25°C) as determined by RT-qPCR. Data is represented as mean ± SEM and was normalized to *cdc-42* mRNA. *n* = 3 independent biological samples, ns, not significant. **(B)** Quantitative analysis of Aβ_1–42_ deposits in the head region of wild-type (red), *unc-54p::A*β*_1–42_* (GMC101, black), and *crh-1(syb385); unc-54p::A*β_1–42_ (purple) animals. The quantity is expressed as mean number ± SEM of Aβ deposits/area of the head region. *n* = 10 animals per genotype. *p < 0.05, ****p < 0.0001. **(C)** Representative images of X-34 staining in wild-type (left), *unc-54p::A*β*_1–42_* (GMC101, middle) and *crh-1(syb385); unc-54p::A*β_1–42_ (right) animals. White arrows indicate Aβ_1–42_ reactive deposits (arrows) in the worm head (dotted white line). Scale bar represents 75 μm.

Next, we asked whether loss of circ-*crh-1* expression reduced Aβ-aggregate deposits in *unc-54::A*β_1–42_ animals, which results in age-progressive paralysis and a shortened lifespan (Figure 1). In order to rule out the effects of circ-*crh-1* loss on the transcription of Aβ_1–42_ expression rather than simply reducing Aβ aggregates in muscle, we first used RT-qPCR analysis to measure Aβ mRNA levels at different aging time-points. We found no significant differences in Aβ gene expression between *unc-54::A*β_1–42_ and *crh-1(syb385)*;*unc-54::A*β_1–42_ animals at 1, 2, and 3-day old adulthood at the higher permissive 25°C temperature (Figure 3A). We then utilized the sensitive amyloid-binding dye X-34 (Link et al., 2001) to specifically stain and visualize *in vivo* Aβ aggregates in muscle cells of *C. elegans* as reported (McColl et al., 2012) and assessed whether circ-*crh-1(-)* mutations reduce the number of Aβ aggregates puncta in *unc-54::A*β_1–42_ expressing worms. Consistent with the delayed onset of the age-progressive Aβ-induced paralysis and restoration of the shortened lifespan, we found that the number of X-34 positive Aβ-aggregates were significantly reduced (*p* < 0.05) in *crh-1(syb385)* mutants carrying *unc-54::A*β_1–42_ compared to GMC101 in 5-day old adults at the higher permissive 25°C temperature (Figures 3B, C). Thus, loss of circ-*crh-1* expression inhibits the accumulation of Aβ aggregates.

### 3.5 circ-*crh-1* expression is required in muscle for Aβ-induced paralysis

**FIGURE 4:**
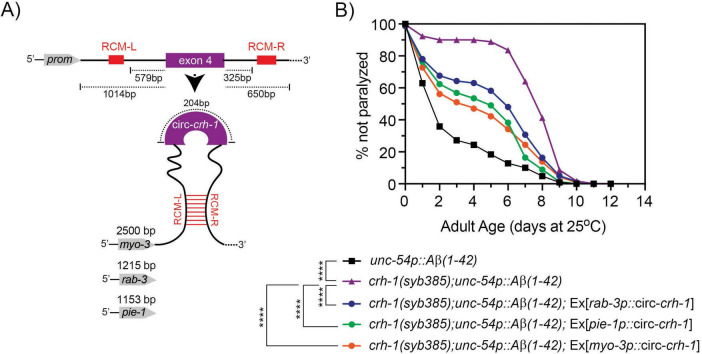
Expression of circ-*crh-1* in multiple tissues can partially rescue the delayed onset of Aβ-induced paralysis in circ-*crh-1* mutants. **(A)** Schematic of plasmid-based minigene used to overexpress circ-*crh-1* under control of tissue-defined promoters. Shown is the *crh-1*(exon4) and reverse complementary match sequences (RCM-L and RCM-R) predicted to facilitate back-splicing of circ-*crh-1*. Promoters and lengths used are *myo-3* with 2,500 bp for muscle expression, *rab-3* with 1,215 bp for pan-neural expression, and *pie-1* with 1,153 bp for germline expression. **(B)** Paralysis measured in *crh-1(syb385); unc-54p::A*β*_1–42_* animals overexpressing circ-*crh-1* in *myo-3*-expressing muscle cells, *rab-3*-expressing neurons, and *pie-1*-expressing germline cells compared to *unc-54p::A*β*_1–42_* (GMC101, black), *crh-1(syb385); unc-54p::A*β*_1–42_* (purple) animals after a temperature upshift from 20°C to 25°C. 3 independent trials with *n* > 140 animals for each assay in the presence of 0.5 μM FUdR. Asterisks indicate statistical significance with ****p < 0.0001.

We next asked whether circ-*crh-1* expression in muscle could restore the delayed onset of Aβ-induced paralysis of *crh-1(syb385)* mutants expressing Aβ_1–42_ in muscle cells. To investigate this question, we used tissue-specific rescue experiments. We previously showed that circ-*crh-1* expression in neurons is an important determinant for lifespan regulation (Knupp et al., 2022). We decided to create *crh-1(syb385); unc-54::A*β_1–42_ transgenic animals that express circ-*crh-1* under select tissue-specific promotors. We cloned the *crh-1*(exon 4) circularizing sequence in between the left and right reverse complementary match (RCM) sequences, and used muscle (*myo-3*), pan-neural (*rab-3*), or germline (*pie-1*) specific promoters to drive the circ-*crh-1* expression transgene (Figure 4A). Expression of circ-*crh-1* under control of the muscle-specific *myo-3* promoter could partially restore the delayed onset of Aβ-induced paralysis of *crh-1(syb385); unc-54::A*β_1–42_ animals at the higher permissive 25°C temperature (Figure 4B), suggesting that circ-*crh-1* expression in muscle is an important determinant for Aβ-induced toxicity. Surprisingly, however, circ-*crh-1* expression driven by *rab-3* and *pie-1* promoters also partially restored Aβ-induced paralysis at 25°C (Figure 4B). These results suggest that in addition to muscle, circ-*crh-1* expression may have additional requirements in other tissues to alter Aβ-induced paralysis.

### 3.6 The delayed onset of Aβ-induced paralysis in circ-*crh-1* mutants is dependent on *col-49* expression

**FIGURE 5:**
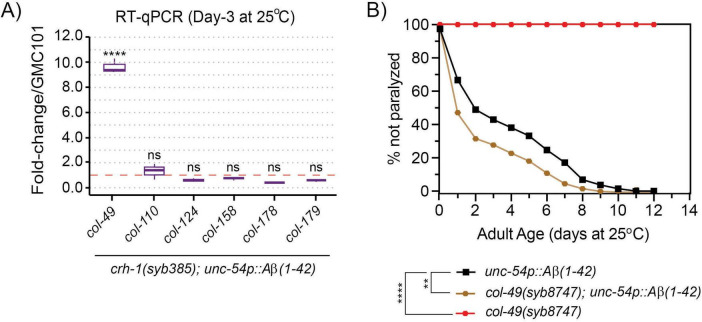
circ-*crh-1* mutations increase *col-49* expression after Aβ-induction, whereas loss of *col-49* promotes Aβ-induced paralysis. **(A)** RT-qPCR expression of 6 predicted cuticular collagen genes in *crh-1(syb385); unc-54p::A*β*_1–42_* normalized to *unc-54p::A*β*_1–42_* (GMC101) at 25°C (after Aβ-induction) in 3-day old adults. *col-49* expression is strongly increased after Aβ-induction in *crh-1(syb385)* mutants. Data is represented as mean ± SEM and was normalized to *act-1* mRNA. *n* = 3 independent biological samples. ns, not significant. ****p < 0.0001. **(B)** Paralysis measured in *unc-54p::A*β*_1–42_* animals carrying a *col-49(syb8747)* mutation (brown) compared to *unc-54p::A*β*_1–42_* (GMC101, black) and *col-49(syb8747)* mutant animals (red). The *syb8747* allele has a 1,180 bp deletion generated by CRISPR/Cas9 (see section “2 Materials and methods”). 3 independent trials with *n* > 140 animals for each assay in the presence of 0.5 μM FUdR. Asterisks indicate statistical significance with **p < 0.01 and ****p < 0.0001.

We previously showed that circ-*crh-1*(-) mutants exhibit widespread transcriptomic changes that might impact various age-related pathways (Cortés-López et al., 2018). Notably, among the identified genes, a subset included collagen-encoding genes with many of which showed increased expression levels in circ-*crh-1*(-) mutants. Interestingly, cuticular collagens are implicated in Aβ aggregate formation and clearance pathways of Aβ in *C. elegans* (Jongsma et al., 2023). We hypothesized that the increased expression of cuticular collagen genes contributes to the amelioration of Aβ-induced toxicity in circ-*crh-1*(-) mutants carrying the *unc-54::A*β*_1–42_* transgene. To test this hypothesis, we selected six cuticular collagen genes of interest from the 21 collagen genes with elevated expression previously identified in *crh-1(syb385)* mutants (Knupp et al., 2022), including collagens that have a known association with lifespan such as *col-49* and *col-179* (Palani et al., 2023). Among the collagen genes tested by RT-qPCR analysis, only *col-49* showed elevated gene expression levels in *crh-1(syb385)* mutants expressing *unc-54::A*β*_1–42_* compared to GMC101 controls at 25°C in 3-day old adults (Figure 5A). No detectable differences were observed in total collagen levels in *crh-1(syb385)* mutants compared to wild-type under different cultivation temperatures (Supplementary Figure 2). Thus, the elevated expression of *col-49* in *crh-1(syb385)* mutants likely represents a specific response to Aβ-induced toxicity, rather than a general increase in collagen production driven by circ-*crh-1.*

We next generated *col-49* deletion mutants using a CRISPR/Cas9 strategy and crossed the mutant with GMC101 to assess Aβ-induced toxicity through a paralysis assay at 25°C. We found that *col-49(syb8747); unc-54::A*β*_1–42_* animals exhibited an exacerbated, age-progressive paralysis compared to the control (GMC101) at the higher 25°C permissive temperature (Figure 5B). *col-49(syb8747)* mutants without the *unc-54::A*β*_1–42_* transgene did not display paralysis at 25°C (Figure 5B).

**FIGURE 6:**
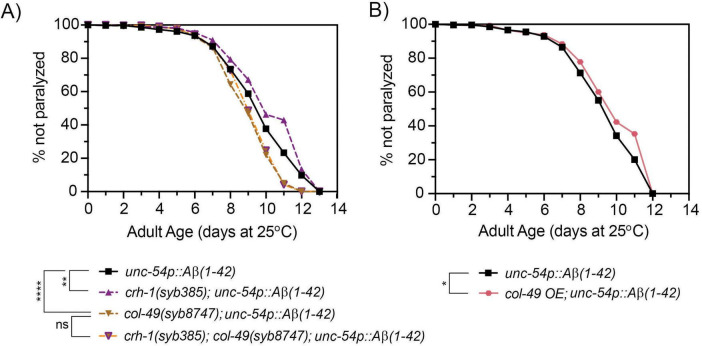
A mutation in *col-49* suppresses the delayed Aβ-induced paralysis of circ-*crh-1* mutants, while *col-49* overexpression reduces Aβ-induced paralysis. **(A)** Paralysis measured in *unc-54p::A*β*_1–42_* animals carrying both *col-49(syb8747)* and *crh-1(385)* mutations (orange line with purple triangle) compared to *unc-54p::A*β*_1–42_* (GMC101, black), *col-49(syb8747); unc-54p::A*β*_1–42_* (light brown) and *crh-1(syb385); unc-54p::A*β*_1–42_* mutant animals (purple). **(B)** Paralysis measured in transgenic animals overexpressing (OE) the *col-49* gene (salmon) compared to *unc-54p::A*β*_1–42_* animals (GMC101, black). **(A,B)** In these experiments, 1-day old adults were upshifted from the non-restrictive 20°C temperature to the higher permissive 25°C temperature to induce paralysis by Aβ. 3 independent trials with *n* > 100 animals for each assay in the presence of 0.5 μM FUdR. ns, not significant. Asterisks indicate statistical significance with *p < 0.05, **p < 0.01 and ****p < 0.0001 (Mantel-Cox log-rank test).

To test whether *col-49* expression is required for the delayed onset of Aβ-induced toxicity observed in *crh-1(syb-385)* mutants, we crossed the *col-49(syb8747)* mutation into *crh-1(syb385)* mutants, both of which carried the *unc-54::A*β*_1–42_* transgene. Mutations in *col-49(syb8747)* significantly suppressed the delayed Aβ-induced paralysis phenotype of *crh-1(syb385)* animals with the *unc-54::A*β*_1–42_* transgene when 1-day old adults were shifted from 20°C to the higher 25°C permissive temperature (Figure 6A). We also examined overexpression (OE) of *col-49* on Aβ-induced paralysis, and found that *col-49* OE transgenic animals weakly but significantly (*p* < 0.05) delayed the onset of Aβ-induced paralysis (Figure 6B) similar as circ-*crh-1(-)* mutants. Collectively, these results suggest that *col-49* gene expression is elevated in circ-*crh-1*(-) mutants, and that loss of circ-*crh-1* promotes Aβ-induced paralysis in a *col-49* dependent manner.

## 4 Discussion

The expression of circRNAs accumulate during aging in *C. elegans*, *Drosophila* and mice (Westholm et al., 2014; Gruner et al., 2016; Hall et al., 2017; Cortés-López et al., 2018) as well as in age-associated disorders such as Alzheimer’s disease (AD) (Dube et al., 2019), but the role of age-accumulated circRNAs in AD remains unclear. We assessed whether loss of a single abundant and age-accumulated circRNA, called circ-*crh-1*, could protect against amyloid β-induced toxicity using a well-established *C. elegans* transgenic strain that expresses human Aβ_1–42_ in muscle cells, which results in age-progressive full body paralysis and shortened lifespan. We found a significant delay in the onset of Aβ-induced paralysis in two independent circ-*crh-1*(-) mutants, which could partially be rescued by re-introducing circ-*crh-1* expression in muscle, neurons and germline cells. In addition, we observed that circ-*crh-1*(-) mutants improve the reduced lifespan of muscle expressing Aβ_1–42_ animals. We further demonstrated that genetic removal of circ-*crh-1* expression results in a reduction of Aβ aggregates, suggesting that loss of a single age-accumulated circRNA protects against the age-related onset of Aβ toxicity in *C. elegans*.

Our transgenic experiments implicate circ-*crh-1* expression in muscle by which circ-*crh-1* could delay the onset of Aβ_1–42_-induced paralysis in the GMC101 strain. We also observed that circ-*crh-1* expression in neurons and germline cells could partially restore the delayed onset of Aβ_1–42_-induced paralysis caused by circ-*crh-1* mutations. The GMC101 strain is an inducible model for muscle expressing Aβ_1–42_ aggregation and proteotoxicity (McColl et al., 2012). Aβ peptides can spread and transfer between cells (Domert et al., 2014). In *C. elegans*, intracellular Aβ_1–42_ peptides expressed in a subset of neurons are able to spread to other cells and distal tissues, and targeted depletion of neuronal Aβ can systemically delay Aβ aggregation (Gallrein et al., 2021). Our rescue experiments suggest that circ-*crh-1* expression is required in other tissues besides muscle for Aβ_1–42_-induced paralysis, but it remains uncertain whether the GMC101 strain exhibits systemic defects and aggregation of Aβ_1–42_ beyond the tissue of expression (i.e., muscle). It might also be possible that overexpression of circ-*crh-1* in neurons and germline cells leads to non-cell-autonomous rescue of muscle expressing Aβ_1–42_ aggregates (Nussbaum-Krammer and Morimoto, 2014). Further experiments demonstrating *in vivo* expression of circ-*crh-1* coupled with labeling Aβ_1–42_ could offer insight into the mechanisms through which circ-*crh-1* regulates Aβ_1–42_ aggregation and its phenotypic consequences.

Using transcriptome-wide analysis, we previously showed increased expression of multiple collagen-encoding genes in circ-*crh-1*(-) mutants (Knupp et al., 2022). Collagens have previously been linked to Alzheimer’s disease, with several collagens influencing Aβ-aggregate formation (Cheng et al., 2009; Tong et al., 2010). Moreover, a recent study reported that *C. elegans* cuticular collagens are implicated in extracellular Aβ-aggregate formation and clearance (Jongsma et al., 2023). We selected 6 collagen genes with elevated expression in circ-*crh-1*(-) mutants (Knupp et al., 2022) and tested them in circ-*crh-1*(-) mutants expressing Aβ_1–42_ in muscle. We found that expression levels of the predicted cuticular collagen, *col-49*, with a known role in lifespan regulation (Palani et al., 2023) and cuticular integrity (Jackson et al., 2014) was significantly increased, while the other tested collagen genes were not different from the GMC101 control. We do not yet know how loss of circ-*crh-1* expression results in increased *col-49* mRNA levels in the presence of muscle expressing Aβ_1–42_. We favor the possibility that circ-*crh-1* interacts with RNA-binding proteins (RBPs) to regulate their expression and function by acting as a sponge, decoy, scaffold or recruiter, which could affect the fate of mRNA targets of RBPs through post-transcriptional processes (Patop et al., 2019; Chen, 2020). In this scenario, and consistent with loss of circ-*crh-1* expression resulting in transcriptomic changes (Knupp et al., 2022), circ-*crh-1* may sequester away RBPs from *col-49* mRNA targets, which in turn alters its expression.

Collagen biosynthesis and stability in *C. elegans* can affect Aβ-aggregate levels (Jongsma et al., 2023). While we did not observe any changes in overall collagen levels in whole circ-*crh-1*(-) mutant animals, we found that mutants lacking *col-49* exacerbate the Aβ_1–42_-induced paralysis. This exacerbation might be explained by circ-*crh-1* indirectly modulating *col-49* mRNA levels through one or more yet unidentified RBPs, thereby altering Aβ-induced toxicity. Consistent with this hypothesis, our findings show that *col-49* expression is elevated in circ-*crh-1(-)* mutants. We further demonstrated that the delayed onset of Aβ-induced paralysis observed in circ-*crh-1(-)* mutants is dependent on *col-49* expression, as mutations in *col-49* can suppress the delayed onset of Aβ-induced paralysis of circ-*crh-1* mutants. Further research on identifying the specific RBP(s) that interact with circ-*crh-1* could provide crucial insights into the regulatory mechanisms by which the loss of circ-*crh-1* promotes Aβ_1–42_-induced paralysis.

In conclusion, our study shows that the expression of *crh-1* circRNAs is important for modulating Aβ-induced toxicity in Alzheimer’s disease (AD) and could pave the way for using *C. elegans* to study circRNAs in AD and its relationship to aging.

## Data Availability

The original contributions presented in this study are included in this article/Supplementary material, further inquiries can be directed to the corresponding author.

## Supplementary material

Supplementary Figure 1 Lifespan analysis of GMC101 at 20°C. Lifespan curve for *unc-54p::A*β*_1–42_* (GMC101) animals compared to wild-type animals at 20°C. There is a non-significant difference in mean lifespan between *unc-54p::A*β*_1–42_* and wild-type controls (*p* = 0.642, Mantel-Cox log-rank test). See Supplementary Table 3 for lifespan statistics. *n* = 4 independent lifespan assays were performed with *n* = 90-120 animals for each assay and genotype in the presence of 0.5 μM FUdR (see section “2 Materials and methods”).

Supplementary Figure 2 Total collagen in circ-*crh-1* mutants at different temperatures. Total collagen-to-protein ratio in *crh-1(syb385)* mutants compared to wild-type for 1-day adult worms at 15°C, 20°C, and 25°C. There is a non-significant difference in mean total collagen level between *crh-1*(*syb385*) mutants and wild-type animals (15°C, *p* = 0.423; 20°C, *p* = 0.632; 25°C, *p* = 0.250). *n* = 3 independent total collagen determination assays.

## References

[B1] Alshareef H. Ballinger T. Rojas E. Van Der Linden A. M. (2024). Loss of age-accumulated crh-1 circRNAs ameliorate amyloid β-induced toxicity in a C. Elegans model for Alzheimer’s disease. *bioRxiv [Preprint]* 10.3389/fnagi.2025.1464015 PMC11973312 40196178

[B2] Beylerli O. Beilerli A. Ilyasova T. Shumadalova A. Shi H. Sufianov A. (2024). CircRNAs in Alzheimer’s disease: What are the prospects? *Noncoding RNA Res.* 9 203–210. 10.1016/j.ncrna.2023.11.011 38125754 PMC10730436

[B3] Brenner S. (1974). The genetics of Caenorhabditis elegans. *Genetics* 77 71–94. 10.1093/genetics/77.1.71 4366476 PMC1213120

[B4] Chen L. (2020). The expanding regulatory mechanisms and cellular functions of circular RNAs. *Nat. Rev. Mol. Cell. Biol.* 21 475–490. 10.1038/s41580-020-0243-y 32366901

[B5] Cheng J. Dubal D. Kim D. Legleiter J. Cheng I. Yu G. (2009). Collagen VI protects neurons against Abeta toxicity. *Nat. Neurosci.* 12 119–121. 10.1038/nn.2240 19122666 PMC2812922

[B6] Cortés-López M. Gruner M. Cooper D. Gruner H. Voda A. van der Linden A. (2018). Global accumulation of circRNAs during aging in Caenorhabditis elegans. *BMC Genomics* 19:8. 10.1186/s12864-017-4386-y 29298683 PMC5753478

[B7] Domert J. Rao S. Agholme L. Brorsson A. Marcusson J. Hallbeck M. (2014). Spreading of amyloid-β peptides via neuritic cell-to-cell transfer is dependent on insufficient cellular clearance. *Neurobiol. Dis.* 65 82–92. 10.1016/j.nbd.2013.12.019 24412310

[B8] Dube U. Del-Aguila J. Li Z. Budde J. Jiang S. Hsu S. (2019). An atlas of cortical circular RNA expression in Alzheimer disease brains demonstrates clinical and pathological associations. *Nat. Neurosci.* 22 1903–1912. 10.1038/s41593-019-0501-5 31591557 PMC6858549

[B9] Fay D. (2006). Genetic mapping and manipulation: Chapter 1–Introduction and basics. *WormBook* 1–12. 10.1895/wormbook.1.90.1 18050463 PMC4780916

[B10] Gallrein C. Iburg M. Michelberger T. Koçak A. Puchkov D. Liu F. (2021). Novel amyloid-beta pathology C. elegans model reveals distinct neurons as seeds of pathogenicity. *Prog. Neurobiol.* 198:101907. 10.1016/j.pneurobio.2020.101907 32926945

[B11] Gruner H. Cortés-López M. Cooper D. Bauer M. Miura P. (2016). CircRNA accumulation in the aging mouse brain. *Sci. Rep.* 6:38907. 10.1038/srep38907 27958329 PMC5153657

[B12] Hall H. Medina P. Cooper D. Escobedo S. Rounds J. Brennan K. (2017). Transcriptome profiling of aging Drosophila photoreceptors reveals gene expression trends that correlate with visual senescence. *BMC Genomics* 18:894. 10.1186/s12864-017-4304-3 29162050 PMC5698953

[B13] Han S. Lee D. Lee H. Kim D. Son H. Yang J. (2016). OASIS 2: Online application for survival analysis 2 with features for the analysis of maximal lifespan and healthspan in aging research. *Oncotarget* 7 56147–56152. 10.18632/oncotarget.11269 27528229 PMC5302902

[B14] Hansen T. Jensen T. Clausen B. Bramsen J. Finsen B. Damgaard C. (2013). Natural RNA circles function as efficient microRNA sponges. *Nature* 495 384–388. 10.1038/nature11993 23446346

[B15] Jackson B. Abete-Luzi P. Krause M. Eisenmann D. (2014). Use of an activated beta-catenin to identify Wnt pathway target genes in caenorhabditis elegans, including a subset of collagen genes expressed in late larval development. *G3 (Bethesda)* 4 733–747. 10.1534/g3.113.009522 24569038 PMC4059243

[B16] Jeck W. Sorrentino J. Wang K. Slevin M. Burd C. Liu J. (2013). Circular RNAs are abundant, conserved, and associated with ALU repeats. *RNA* 19 141–157. 10.1261/rna.035667.112 23249747 PMC3543092

[B17] Jongsma E. Goyala A. Mateos J. Ewald C. (2023). Removal of extracellular human amyloid beta aggregates by extracellular proteases in C. elegans. *Elife* 12:e83465. 10.7554/eLife.83465 37728486 PMC10541181

[B18] Kim E. Kim Y. Lee S. (2021). Emerging functions of circular RNA in aging. *Trends Genet.* 37 819–829. 10.1016/j.tig.2021.04.014 34016449

[B19] Knupp D. Miura P. (2018). CircRNA accumulation: A new hallmark of aging? *Mech. Ageing Dev.* 173 71–79. 10.1016/j.mad.2018.05.001 29753875 PMC6191176

[B20] Knupp D. Jorgensen B. Alshareef H. Bhat J. Grubbs J. Miura P. (2022). Loss of circRNAs from the crh-1 gene extends the mean lifespan in Caenorhabditis elegans. *Aging Cell.* 21:e13560. 10.1111/acel.13560 35102684 PMC8844124

[B21] Li X. Yang L. Chen L. (2018). The biogenesis, functions, and challenges of circular RNAs. *Mol. Cell.* 71 428–442. 10.1016/j.molcel.2018.06.034 30057200

[B22] Link C. Johnson C. Fonte V. Paupard M. Hall D. Styren S. (2001). Visualization of fibrillar amyloid deposits in living, transgenic Caenorhabditis elegans animals using the sensitive amyloid dye, X-34. *Neurobiol. Aging* 22 217–226. 10.1016/s0197-4580(00)00237-2 11182471

[B23] McColl G. Roberts B. Pukala T. Kenche V. Roberts C. Link C. (2012). Utility of an improved model of amyloid-beta (Aβ*1*−*42*) toxicity in Caenorhabditis elegans for drug screening for Alzheimer’s disease. *Mol. Neurodegener.* 7:57. 10.1186/1750-1326-7-57 23171715 PMC3519830

[B24] Nussbaum-Krammer C. Morimoto R. (2014). Caenorhabditis elegans as a model system for studying non-cell-autonomous mechanisms in protein-misfolding diseases. *Dis. Model. Mech.* 7 31–39. 10.1242/dmm.013011 24396152 PMC3882046

[B25] Palani S. Sellegounder D. Wibisono P. Liu Y. (2023). The longevity response to warm temperature is neurally controlled via the regulation of collagen genes. *Aging Cell.* 22:e13815. 10.1111/acel.13815 36895142 PMC10186602

[B26] Patop I. Wüst S. Kadener S. (2019). Past, present, and future of circRNAs. *EMBO J.* 38:e100836. 10.15252/embj.2018100836 31343080 PMC6694216

[B27] Shi Z. Chen T. Yao Q. Zheng L. Zhang Z. Wang J. (2017). The circular RNA ciRS-7 promotes APP and BACE1 degradation in an NF-κB-dependent manner. *FEBS J.* 284 1096–1109. 10.1111/febs.14045 28296235

[B28] Sun F. Zhang Y. Wu X. Xu X. Zhu C. Huang W. (2023). Breviscapine combined with BMSCs reduces Aβ deposition in rat with Alzheimer’s disease by regulating circular RNA ciRS-7. *Curr. Mol. Med.* 23 76–86. 10.2174/1566524022666220113151044 35048805

[B29] Teuscher A. Statzer C. Pantasis S. Bordoli M. Ewald C. (2019). Assessing collagen deposition during aging in mammalian tissue and in Caenorhabditis elegans. *Methods Mol. Biol.* 1944 169–188. 10.1007/978-1-4939-9095-5_13 30840243 PMC6457433

[B30] Tong Y. Xu Y. Scearce-Levie K. Ptácek L. Fu Y. (2010). COL25A1 triggers and promotes Alzheimer’s disease-like pathology in vivo. *Neurogenetics* 11 41–52. 10.1007/s10048-009-0201-5 19548013 PMC2807601

[B31] Weigelt C. Sehgal R. Tain L. Cheng J. Eßer J. Pahl A. (2020). An insulin-sensitive circular RNA that regulates lifespan in Drosophila. *Mol. Cell.* 79 268–279.e5. 10.1016/j.molcel.2020.06.011 32592682 PMC7318944

[B32] Westholm J. Miura P. Olson S. Shenker S. Joseph B. Sanfilippo P. (2014). Genome-wide analysis of drosophila circular RNAs reveals their structural and sequence properties and age-dependent neural accumulation. *Cell. Rep.* 9 1966–1980. 10.1016/j.celrep.2014.10.062 25544350 PMC4279448

[B33] Zhang N. Gao Y. Yu S. Sun X. Shen K. (2020). Berberine attenuates Aβ42-induced neuronal damage through regulating circHDAC9/miR-142-5p axis in human neuronal cells. *Life Sci.* 252:117637. 10.1016/j.lfs.2020.117637 32251633

[B34] Zhang X. Wang H. Zhang Y. Lu X. Chen L. Yang L. (2014). Complementary sequence-mediated exon circularization. *Cell* 159 134–147. 10.1016/j.cell.2014.09.001 25242744

