# Supplementary figures and images for: Loss of age-accumulated crh-1 circRNAs ameliorate amyloid β-induced toxicity in a C. elegans model for Alzheimer’s disease
Source: Front Aging Neurosci. 2025 Mar 24;17:1464015. doi: 10.3389/fnagi.2025.1464015 (PMC11973312; doi:10.3389/fnagi.2025.1464015)

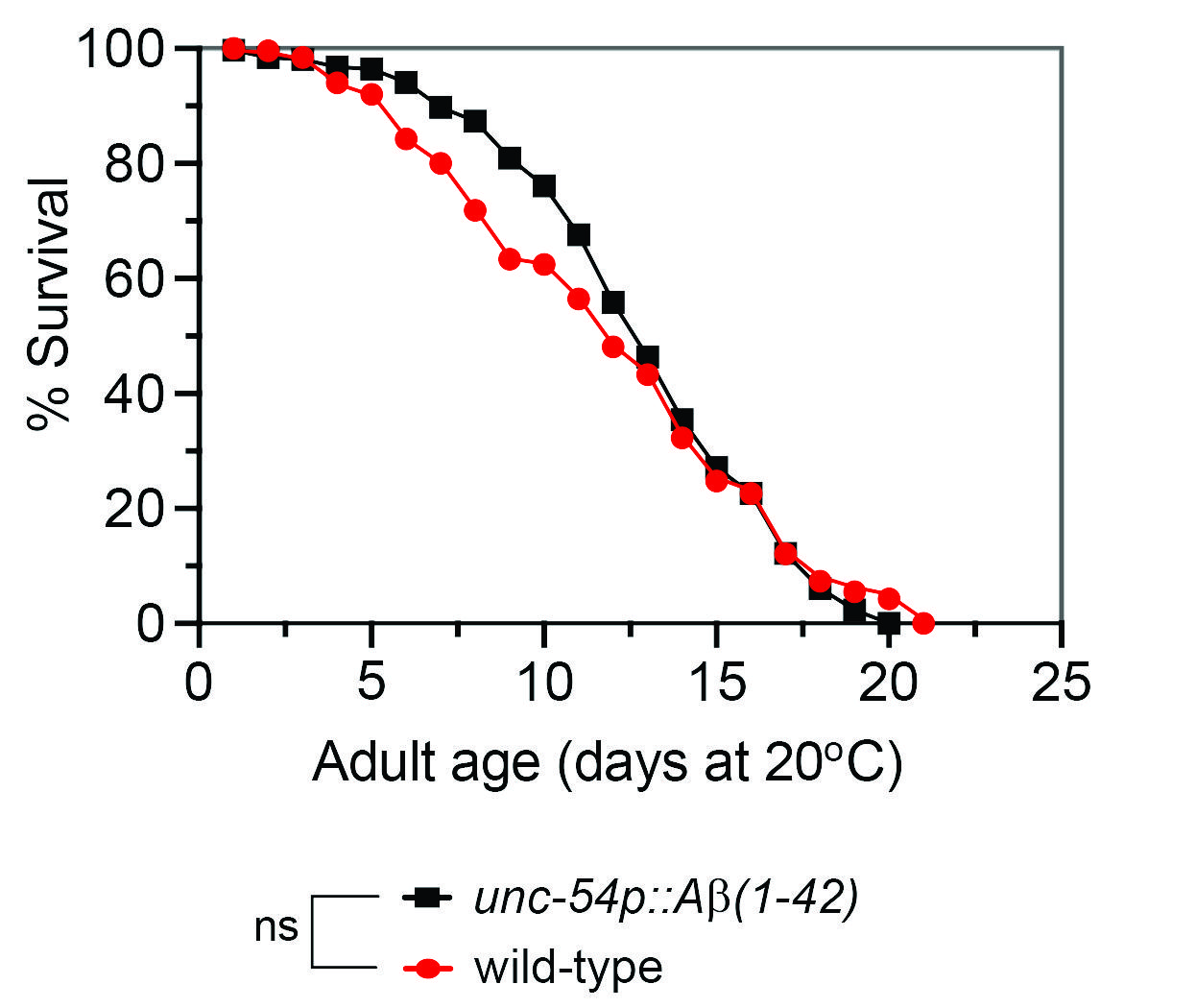

Supplement: Supplementary Figure 1 — Lifespan analysis of GMC101 at 20°C. Lifespan curve for unc-54p::Aβ1–42 (GMC101) animals compared to wild-type animals at 20°C. There is a non-significant difference in mean lifespan between unc-54p::Aβ1–42 and wild-type controls (p = 0.642, Mantel-Cox log-rank test). See Supplementary Table 3 for lifespan statistics. n = 4 independent lifespan assays were performed with n = 90-120 animals for each assay and genotype in the presence of 0.5 μM FUdR (see section “2 Materials and methods”). [file Image_1.JPEG]

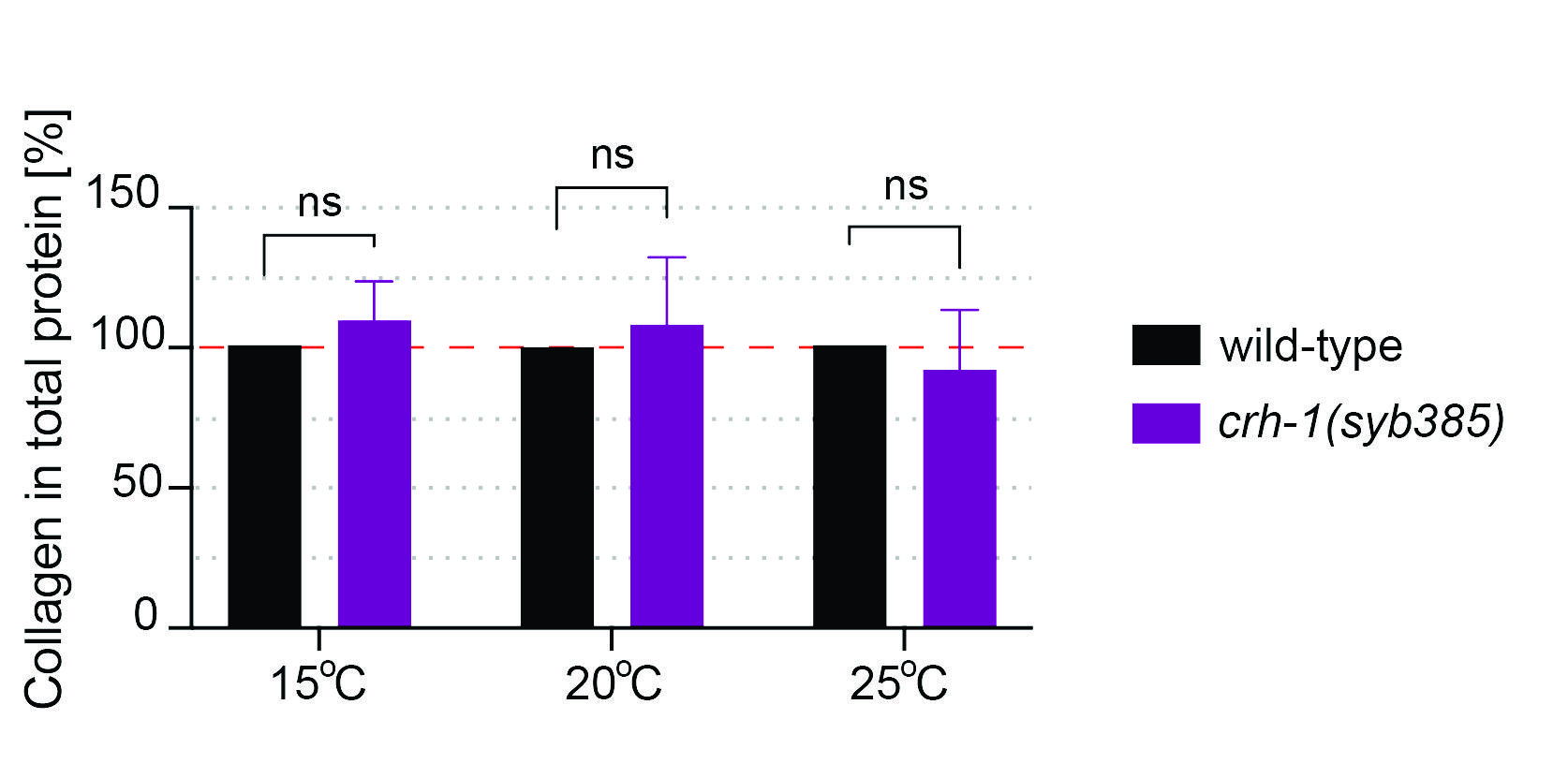

Supplement: Supplementary Figure 2 — Total collagen in circ-crh-1 mutants at different temperatures. Total collagen-to-protein ratio in crh-1(syb385) mutants compared to wild-type for 1-day adult worms at 15°C, 20°C, and 25°C. There is a non-significant difference in mean total collagen level between crh-1(syb385) mutants and wild-type animals (15°C, p = 0.423; 20°C, p = 0.632; 25°C, p = 0.250). n = 3 independent total collagen determination assays. [file Image_2.JPEG]
